# Metabolic dysfunction-associated fatty liver disease and liver function markers are associated with Crohn’s disease but not Ulcerative Colitis: a prospective cohort study

**DOI:** 10.1007/s12072-022-10424-6

**Published:** 2022-10-04

**Authors:** Jie Chen, Lintao Dan, Xinru Tu, Yuhao Sun, Minzi Deng, Xuejie Chen, Therese Hesketh, Ran Li, Xiaoyan Wang, Xue Li

**Affiliations:** 1 Department of Big Data in Health Science, Center of Clinical Big Data and Analytics of the Second Affiliated Hospital, Zhejiang University School of Medicine, Hangzhou, China; 2 Centre for Global Health, Zhejiang University, Hangzhou, China; 3 Department of Gastroenterology, The Third Xiangya Hospital of Central South University, Changsha, China; 4 Institute for Global Health, University College London, London, UK; 5 Centre for Global Health, Usher Institute, University of Edinburgh, Edinburgh, UK

**Keywords:** Metabolic dysfunction-associated fatty liver disease, Inflammatory bowel disease, Liver function biomarkers, Cohort study, UK Biobank, MAFLD, CD, UC, IBD, Survival analysis

## Abstract

**Background:**

Metabolic dysfunction-associated fatty liver disease (MAFLD) is recently recognized as a condition featured with metabolic dysfunctions in liver. It has been supposed that MAFLD might contribute to the development of IBD, but evidence from prospective cohort studies is lacking and inconclusive.

**Methods:**

A total of 221,546 females and 183,867 males from the UK Biobank cohort enrolled in 2006–2010 were included to examine whether MAFLD and liver function markers were related to incident IBD. MAFLD was identified based on hepatic steatosis defined by fatty liver index plus the prevalence of overweight, type 2 diabetes mellitus, or at least two metabolic abnormalities. Biomarker related to liver function (albumin [ALB], alkaline phosphatase [ALP], alanine transaminase [ALT], aspartate transaminase [AST]; gamma-glutamyl transferase [GGT], total bilirubin [TB], total protein [TP]) was measured using colorimetric or enzymatic assays. The incidence of IBD was ascertained based on primary care and inpatient records. Cox proportional hazard model was used to estimate hazard ratios (HRs) with 95% confidence intervals (CI) for the magnitude of their associations.

**Results:**

With a mean follow-up of 12.1 years, 2228 incident IBD cases were documented. We identified 150,385 individuals with MAFLD at baseline and 86% participants’ circulating liver function markers were within the normal range. Participants with MAFLD were associated with a 12% (HR 1.12, 95% CI 1.03, 1.23, *p* = 0.012) increased risk of IBD compared with those without MAFLD at baseline; the association was stronger (*p*-_Heterogeneity_ = 0.006) with Crohn's disease (HR 1.35, 95% CI 1.15, 1.59, *p* < 0.001) than ulcerative colitis (HR 1.03, 95% CI 0.93, 1.15, *p* = 0.57). As for the serum liver function markers, the HRs of IBD for per 1-SD increment in ALB, ALP, AST, and TB concentration were 0.86 (95% CI 0.83, 0.90, *p* < 0.001), 1.18 (95% CI 1.13, 1.24, *p* < 0.001), 0.95 (95% CI 0.91, 0.99, *p* = 0.027), 0.92 (95% CI 0.87, 0.96, *p* < 0.001), respectively. We did not observe significant associations of GGT and TP with IBD.

**Conclusions:**

Individuals with MAFLD were at increased risk of developing IBD, especially CD, but not UC. Circulating levels of liver function biomarkers as the surrogate indicators of MAFLD were also associated with IBD risk.

**Supplementary Information:**

The online version contains supplementary material available at 10.1007/s12072-022-10424-6.

## Introduction

Inflammatory bowel disease (IBD), comprising Crohn's disease (CD) and ulcerative colitis (UC), is a lifelong chronic disease posing a substantial burden on individuals, families and health systems [1]. Previous reviews have indicated that metabolic dysfunction can lead to the incidence of IBD by molecular-level connections to loss of barrier integrity, intestinal pro-inflammatory state, and alterations in gut microbiota [2]. Data from the Nurses’ Health Study demonstrated women with obesity, as a condition of metabolic dysfunction, have more than doubled risk of CD compared to healthy women [3]. Low high-density lipoprotein cholesterol level, as a predictor of metabolic dysfunction, has been found to be associated with subsequent IBD diagnosis in a Finnish study consisting of 3551 children and adolescents [4]. Identifying the components of metabolic dysfunction contributing to the development of IBD would have clinical and practical implications to formulate intervention strategies to reduce IBD burden among high-risk populations.

An international consensus group recently proposed metabolic dysfunction-associated fatty liver disease (MAFLD), highlighting that liver disease clinically presents with symptoms of metabolic dysfunctions [5]. There is increasing evidence showing that individuals with MAFLD have higher prevalent risk of IBD than general population and those with comorbidity of MAFLD and IBD are predominant with clinical features of metabolic disorders. However, current studies on the comorbidity of IBD and MAFLD are limited and with few prospective evidence. Data from a cross-sectional study including 2549 Spanish showed a significantly higher prevalence of MAFLD among individuals with IBD than the general population (42% vs. 32%) [6]. But the direction of the association could not be clarified due to the cross-sectional design. It remains unknown whether MAFLD has impact on the development of IBD. Meanwhile, it is necessary to take the chronologic order of development of these two diseases into account.

Here, we performed a prospective cohort study to evaluate the association of MAFLD with incident IBD. In addition, we investigated the association between circulating liver function biomarkers with incident IBD to further explore the impact liver function on IBD.

## Material and methods

### Study population

This study leveraged all participant-specific data from the UK Biobank. The UK Biobank is an ongoing national prospective cohort project that enrolled over 500,000 volunteers from 22 assessment centers in the UK between 2006 and 2010. At baseline, each participant would receive a touch-screen questionnaire, a brief computer-assisted interview, physical measurements, and laboratory tests [7]. The UK Biobank also enables the follow-up of medical or health-related records of individuals throughout the UK. Primary care data and inpatient data were extracted from the national data providers in England, Scotland and Wales [8]. All participants have signed an electronic consent, and the North West–Haydock Research Ethics Committee granted ethical approval to use the UK Biobank database (REC reference: 16/NW/0274).

In this study, we excluded (1) participants without sufficient information to evaluate MAFLD status (*n* = 50,144) or with missing data on the circulating levels of liver function biomarkers (*n* = 22,064); (2) participants with an IBD diagnosis at baseline (*n* = 5537) or during the first year of follow-up (*n* = 139), considering the mean delay of the diagnosis of IBD was 11 months in the UK [9]; (3) and participants with any liver function biomarker outside the normal distribution (*n* = 19,163) based on the Rosner's generalized extreme studentized deviate test [10]. After exclusions, 405,413 participants were eligible to be included in our analysis (Fig. S1). This study followed the Strengthening the Reporting of Observational Studies in Epidemiology (STROBE) guideline.

### Identification of individuals with MAFLD

According to the international expert consensus [11], individuals with MAFLD are defined as individuals with hepatic steatosis plus any of the following conditions: being overweight or obese (i.e., BMI > 25 kg/m^2^), type 2 diabetes mellitus, or at least two metabolic abnormalities. As there were no available liver imaging or histology data in the UK Biobank database, the fatty liver index was used to confirm the presence of hepatic steatosis (fatty liver index ≥ 60) in the baseline population [12]. The fatty liver index is a commonly utilized invasive indicator for hepatic steatosis [13] and has been used for identification of hepatic steatosis in previous studies conducted in the UK Biobank [14, 15]. A cut-off value of fatty liver index ≥ 60 gives a specificity of 91% and a positive likelihood ratio of 5.10 in external validation of the fatty liver index in UK using ^1^H-MRS measures of liver fat [16]. For the diagnosis of type 2 diabetes mellitus, participants (1) with hemoglobin A1C > 47 mmol/mol; (2) with 10th international disease classification (ICD) codes of E11.0-E11.9 or ICD-9 code of 250; or (3) regularly took anti-diabetic medications were identified. Metabolic abnormalities included increased waist circumference, arterial hypertension, hypertriglyceridemia, low high-density lipoprotein cholesterol, prediabetes, insulin resistance, and subclinical inflammation. Detailed definitions are shown in Table S1. Finally, 150,385 individuals were identified with MAFLD at baseline, and 4561 were reassessed same items in the UK Biobank after a median interval of 4.4 years, with 79.8% still retaining this status. The prevalence of MAFLD (37.2%) in our study is comparable to the prevalence (33%) in the general population reported in previous meta-analysis [17].

For exploratory purposes, we used a clinic-biological score, BAAT score, to evaluate the probability of liver fibrosis in individuals with MAFLD. BAAT score ≥ 2 showed a sensitivity of 0.71 and a specificity of 0.80 for predicting advanced liver fibrosis [18]. Details were presented in Supplementary Methods.

### Measurement of circulating liver function biomarkers

Biomarkers were chosen according to the clinical guideline of American College of Gastroenterology for liver function test [19] and the data availability of the UK Biobank Biomarker Project [20]. Circulating levels of alkaline phosphatase (ALP), alanine transaminase (ALT), aspartate transaminase (AST), and gamma-glutamyl transferase (GGT) were measured using the enzymatic rate method. Circulating levels of albumin (ALB), total bilirubin (TB), and total protein (TP) were measured using the colorimetric method. The measurements were performed on Beckman Coulter AU5800 as previously published [20]. In summary, the average within-laboratory coefficient of variation in quality-control samples ranged between 2.09%–2.13% for ALB, 2.84%–3.08% for ALP, 1.16%–2.91% for ALT, 1.33%–2.13% for AST, 1.44%–2.84% for GGT, 1.48%–1.92% for TB, and 1.09%–1.22% for TP. Besides, an age-adjusted partial correlation was used to examine the correlation between biomarkers [21].

### Ascertainment of IBD

The outcome of interest was the incidence of IBD. Information for incident IBD was ascertained through primary care data (recorded specific diagnostic code that can be converted into ICD-10 code), inpatient data (recorded in ICD-9 or ICD-10 code) and death registry (recorded in ICD-10 code). IBD, as well as its subtypes of CD and UC, were identified based on the ICD-10 codes K50 (CD) and K51 (UC) or ICD-9 codes 555 (CD) and 556 (UC). In addition, the disease extent of CD and UC was identified according to the ICD codes based on the highest available degree of anatomic distribution of inflammation [22]. Disease extent of CD was classified as ileal, colonic, and ileocolonic CD; disease extent of UC was classified as ulcerative proctitis, left-sided UC, and pancolitis. We also identified individuals with CD with perianal disease through ICD codes and the operations and procedures code according to the Office of Population Censuses and Surveys Classification of Interventions and Procedures [22]. Details are presented in Table S2.

### Assessment of covariates

In this study, we used predefined covariates. Information, including age, ethnicity (white people, others), education level (college degree, below college degree), smoking status (never smoked, previous or current smoker), alcohol consumption (none to moderate, heavy), was assessed by self-reported questionnaire at the baseline. We included Townsend deprivation index as measure of material deprivation within the population, which was derived by postcodes of participants in the UK Biobank automatedly [23]. The range for none to moderate alcohol consumption was 0–14 g per day for females and 0–28 g per day for males [24]. Dietary factors were obtained from the UK Biobank food frequency questionnaire, which showed good agreement between reported consumption at recruitment and at the repeat assessment center visit, approximately 4 years later [25]. Based on previous research on dietary quality in the UK Biobank, we categorized dietary factors into healthy or unhealthy diets according to the frequency of major food groups [26]. BMI was calculated using height and weight measured at the physical examination centers. Physical activity was collected using a validated short International Physical Activity Questionnaire and assessed as adequate or inadequate based on the recommendation from the American Heart Association [27]. In addition, we included serum C-reactive protein concentration, Charlson Comorbidity Index (CCI), and self-reported usage of non-steroidal anti-inflammatory drugs. CCI was constructed based on 17 comorbidities with assigned weights associated with ICD codes from inpatient data [28]. The medication information was obtained from the baseline medication information in touch-screen questionnaires and verbal interview, including use of non-steroidal anti-inflammatory drugs, antibiotics, proton-pump inhibitors, hormonal replacement therapy, and oral contraceptive pills. Details about processing the covariates were presented in the Supplementary Methods. If covariate information was missing or recorded as “unknown,” we imputed the median values for continuous variables or applied a most frequently used category for categorical variables.

### Statistical analysis

Characteristics of participants were presented as means (standard deviations [SDs]) for continuous variables and numbers (percentages) for categorical variables. Chi‐squared test and *t* test were used to compare the characteristics of those with and without MAFLD as appropriate. When variables were not normally distributed by Anderson–Darling test, medians with interquartile range were presented and Mann–Whitney U test was used for comparisons. Person-years of follow-up were calculated from the time when the participants were first recruited in the UK Biobank to the time of IBD diagnosis, death, or end of follow-up, whichever occurred first. Cox proportional hazards regression was applied to estimate hazard ratios (HRs) and 95% confidence interval (CIs) for the association of MAFLD and level of circulating liver biomarkers in quintiles with incident IBD [29].

Two multivariable models were constructed with conventional confounders: Model 1 was adjusted for age, sex and ethnicity; model 2 (fully adjusted model) was further adjusted for socioeconomic factors (Townsend deprivation index), lifestyle factors (smoking, alcohol consumption, BMI, physical activity, healthy diet), C-reaction protein, and CCI. BMI and C-reaction protein were not included when investigating the associations between MAFLD and IBD due to overcontrol concerns [14, 15]. The usage of hormonal replacement therapy and oral contraceptive pills was included in the fully adjusted model when investigating associations in females. The proportional hazards assumptions of all Cox models were confirmed by the weighted residual method [30] with the smallest *p* value of 0.13. Heterogeneity across disease subtypes was calculated with a contrast test method to assess whether the exposure-disease association differed among the disease subtypes [31].

For the association between circulating liver biomarkers and incident IBD, we calculated the intraclass correlation coefficient of each biomarker to recalibrate the estimate of association to address regression dilution [32]. To evaluate the potential nonlinear association, we also used restricted cubic splines (RCS) with three knots at the 10th, 50th, and 90th percentiles [33]. The number of knots was determined according to the minimized Akaike information criterion, and the overall significance of the spline curve was tested using the likelihood ratio test.

Subgroup analyses involving stratification by age, sex, alcohol consumption, smoking status, and BMI were also performed to identify the interactive factors. Several sensitivity analyses were used to test the robustness of the results. Based on the fully adjusted models, we further: (1) excluded the incident IBD that occurred in the first 2-year period to reduce the potential reverse causality; (2) excluded individuals with baseline colorectal cancer (identified with ICD-10 code C18, C19, and C20 through cancer registry); (3) excluded participants with concomitant liver diseases (identified with ICD-10 code B16-19, E83.0, E83.1, E88.0, I82.0, K70, K73.2, K73.9, K74.3–5, K75.4, K76.5, K83.0 through inpatient data) other than MAFLD to avoid the confounding effect of other liver diseases [34]; (4) adjusted for the use of non-steroidal anti-inflammatory drugs, antibiotics, or proton-pump inhibitors; (5) refilled the missing values using multiple imputation method [35]; (6) Excluding new-onset individuals with MAFLD identified in the repeat assessment of the UK Biobank; (7) investigated the association between the AST to ALT ratio and incident IBD as it is a common indicator for liver function test; (8) investigated associations of liver function makers with IBD in subgroups stratified by the normal range of these markers; and (9) evaluated the association between individuals with MAFLD with different probability of advanced fibrosis and risk of IBD. R 4.1.1 and SAS 9.4 (SAS Institute). All statistical tests were two-sided, and a *p value* < 0.05 was statistically significant.

## Results

### Characteristics of participants

After a mean follow-up of 12.1 years, we identified 2228 incident IBD cases (685 CD and 1543 UC). Table 1 summarizes the characteristics of the participants, among which 37.1% (*n* = 150,385) were identified with MAFLD at baseline. The mean (SD) age at baseline of the 405,413 participants was 56.6 (8.1), comprising 221, 546 (54.6%) females and 183,867 (45.4%) males. Participants with baseline MAFLD were more likely to be older, male, physically inactive, had an unhealthy diet, and had a higher CCI than participants without MAFLD at baseline (All *p* < 0.001).

**Table 1:** Characteristics of participants^a^

	Overall (*n* = 405,413)	Participants with baseline MAFLD (*n* = 150,385)	Participants without baseline MAFLD (*n* = 255,028)	*P*^*b*^
Age at recruitment, year	56.6 (8.1)	57.4 (7.8)	56.1 (8.2)	< 0.001
Sex (%)				< 0.001
Female	221,546 (54.6)	55,022 (36.6)	166,524 (65.3)	
Male	183,867 (45.4)	95,363 (63.4)	88,504 (34.7)	
Townsend deprivation index	− 2.2 (− 3.7, 0.5)	− 1.9 (− 3.5, 0.9)	− 2.3 (− 3.7, 0.2)	< 0.001
BMI, kg/m^2^	27.4 (4.7)	31.5 (4.3)	24.9 (2.8)	< 0.001
Waist circumstances, cm	90.1 (13.3)	102.6 (9.7)	82.7 (8.9)	
Education (%)				0.62
College and above	131,876 (32.9)	39,984 (27.0)	91,752 (36.4)	
Below college	269,213 (67.1)	108,283 (73.0)	160,624 (63.6)	
Ethnicity (%)				< 0.001
White	382,372 (94.8)	141,719 (94.7)	240,653 (94.8)	
Others	21,171 (5.2)	7,883 (5.3)	13,288 (5.2)	
Physical activity (%)^b^				< 0.001
Adequate	282,805 (69.8)	95,500 (63.5)	187,305 (73.4)	
Inadequate	122,608 (30.2)	54,885 (36.5)	67,723 (26.6)	
Smoking status (%)				< 0.001
Previous or current smokers	181,612 (45.0)	76,958 (51.5)	104,654 (41.2)	
Never smoked	221,804 (55.0)	72,521 (48.5)	149,283 (58.8)	
Alcohol consumption (%)^c^				< 0.001
None to moderate	80,390 (19.9)	34,920 (23.3)	45,470 (17.9)	
Heavy	324,185 (80.1)	115,107 (76.7)	209,078 (82.1)	
Healthy diet (%)				< 0.001
Yes	276,583 (71.9)	90,555 (64.2)	186,028 (76.3)	
No	108,236 (28.1)	50,485 (35.8)	57,751 (23.7)	
Charlson Comorbidity Index, scores^d^	0.3 (0.9)	0.3 (0.9)	0.2 (0.8)	< 0.001
Albumin, g/L	45.2 (43.5, 46.9)	45.1 (43.4, 46.8)	45.3 (43.6, 47.0)	< 0.001
Alkaline phosphatase, U/L	79.9 (67.0, 95.1)	84.1 (71.2, 99.5)	77.4 (64.7, 92.3)	< 0.001
Alanine transaminase, U/L	19.9 (15.3, 26.7)	25.4 (19.6, 33.7)	17.5 (14.0, 22.3)	< 0.001
Aspartate transaminase, U/L	24.2 (20.9, 28.4)	25.9 (22.2, 30.7)	23.3 (20.3, 27.1)	< 0.001
C-reactive protein, mg/L	1.31 (0.65, 2.71)	2.11 (1.1, 4.1)	0.97 (0.5, 1.9)	< 0.001
Gamma-glutamyl transferase, U/L	25.9 (18.4, 39.4)	38.2 (27.4, 56.3)	21.2 (16.3, 29.2)	< 0.001
Total bilirubin, μmol/L	8.03 (6.4, 10.3)	8.03 (6.4, 10.3)	8.0 (6.4, 10.3)	< 0.001
Total protein, g/L	72.3 (69.7, 75.0)	72.6 (70.0, 75.2)	72.09 (69.5, 74.8)	< 0.001

*BMI* body mass index, *MAFLD* metabolic dysfunction-associated fatty liver disease

^a^Mean (SD) values and percentages are reported for continuous and categorical variables, respectively. When variables were not normally distributed, medians with interquartile range are presented

^b^compared between individuals with and without MAFLD

^c^Adequate physical activity was defined as 150 min moderate activity per week, or ≥ 75 min vigorous activity per week, or equivalent combination, or moderate physical activity at least 5 days a week or vigorous activity once a week

^d^None to moderate alcohol consumption was defined as 0–14 g/d for women and 0–28 g/d for men according to US dietary guidelines, above which is defined as heavy level

^e^Charlson Comorbidity Index was constructed based on 17 comorbidities with assigned weights associated with ICD codes from hospital records, ranging from 0 to 16

### MAFLD and risk of incident IBD

The associations between MAFLD and risk of incident IBD and its subtypes are shown in Table 2. Individuals with MAFLD were associated with an increased risk of incident IBD (HR 1.12, 95% CI 1.03, 1.23, *p* = 0.012) compared with those without MAFLD. When investigating the incidence of CD and UC independently, substantial heterogeneity was detected (p-heterogeneity = 0.006). The association between MAFLD and CD incidence was consistent and significant (HR 1.35, 95% CI 1.15, 1.59, *p* < 0.001), while no significant association was observed between MAFLD and UC incidence (HR 1.03, 95% CI 0.93, 1.15, *p* = 0.57). Although no evidence of heterogeneity across the disease extent of CD or UC was observed (All *p*-heterogeneity > 0.05), participants with MAFLD were significantly associated with an increased risk of colonic CD (HR 1.83 95% CI 1.12, 2.97, *p* = 0.015) and ileocolonic or unspecified CD (HR 1.28, 95% CI 1.05, 1.55, *p* = 0.013) (Table 2). For CD with perianal disease, we documented 21 and 18 incident cases among individuals with and without MAFLD, respectively. We found a significant association between MAFLD and CD with perianal disease (HR 2.50, 95% CI 1.26, 4.98, *p* = 0.009).

**Table 2:** Association between MAFLD and risk of incident IBD and its subtypes

	Non-MAFLD (*n* = 255,028, totaling 3,109,082 person-years)	MAFLD (*n* = 150,385, totaling 1,807,156 person years)	*P*	*P*-heterogeneity^a^
Incident IBD				
Cases	1273	955		
HR (95% CI)^b^	Ref	1.22 (1.12, 1.33)	< 0.001	
HR (95% CI)^c^	Ref	1.12 (1.03, 1.23)	0.012	
Subtypes of IBD				0.006
Crohn’s disease				
Cases	377	308		
HR (95% CI)^b^	Ref	1.40 (1.19, 1.64)	< 0.001	
HR (95% CI)^c^	Ref	1.35 (1.15, 1.59)	< 0.001	
Ulcerative colitis				
Cases	896	647		
HR (95% CI)^b^	Ref	1.12 (1.01, 1.25)	0.031	
HR (95% CI)^c^	Ref	1.03 (0.93, 1.15)	0.57	
Disease extent of CD				0.058
Ileal CD				
Cases	74	63		
HR (95% CI)^b^	Ref	1.49 (1.05, 2.12)	0.025	
HR (95% CI)^c^	Ref	1.37 (0.96, 1.96)	0.084	
Colonic CD				
Cases	37	37		
HR (95% CI)^b^	Ref	1.62 (1.01, 2.58)	0.043	
HR (95% CI)^c^	Ref	1.83 (1.12, 2.97)	0.015	
Ileocolonic or unspecific CD				
Cases	266	208		
HR (95% CI)^b^	Ref	1.34 (1.10, 1.63)	0.003	
HR (95% CI)^c^	Ref	1.28 (1.05, 1.55)	0.013	
Disease extent of UC				0.16
Ulcerative proctitis				
Cases	97	50		
HR (95% CI)^b^	Ref	0.75 (0.53, 1.06)	0.098	
HR (95% CI)^c^	Ref	0.75 (0.52, 1.07)	0.11	
Left-sided UC				
Cases	90	57		
HR (95% CI)^b^	Ref	1.05 (0.74, 1.47)	0.79	
HR (95% CI)^c^	Ref	0.89 (0.63, 1.26)	0.51	
Pancolitis				
Cases	53	43		
HR (95% CI)^b^	Ref	1.32 (0.88, 1.98)	0.19	
HR (95% CI)^c^	Ref	1.28 (0.83, 1.96)	0.26	
Unspecific UC				
Cases	656	497		
HR (95% CI)^b^	Ref	1.18 (1.04, 1.33)	0.008	
HR (95% CI)^c^	Ref	1.07 (0.95, 1.21)	0.28	

*CD* Crohn’s disease, *IBD* inflammatory bowel disease, *MAFLD* metabolic dysfunction-associated fatty liver disease, *UC* ulcerative colitis

^a^P for heterogeneity was calculated using the contrast method based on a fully unconstrained approach

^b^adjusted for age, sex, ethnicity

^c^further adjusted for smoking status, physical activity level, alcohol consumption, Townsend deprivation index, healthy diet and Charlson Comorbidity Index

### Circulating levels of liver function biomarkers and risk of incident IBD

About 86% of participants had circulating levels of liver function biomarkers within the normal ranges (Table S3). The age-adjusted partial correlations between these biomarkers are shown in Table S4. Over a median interval of 4.3 years, the reproducibility (intraclass correlation coefficients) during the reassessment of circulating liver function markers among 12,081 participants ranged between 0.46 and 0.77 (Table S5).

The circulating levels of ALB, AST, and TB were inversely associated with IBD, while ALP was positively associated with IBD (Table 3). The multivariable HRs (95% CI) of IBD for each 1-SD increment in ALB, ALP, AST, and TB concentration were 0.86 (95% CI 0.83, 0.90, *p* < 0.001), 1.18 (95% CI 1.13, 1.24, *p* < 0.001), 0.95 (95% CI 0.91, 0.99, *p* = 0.027), 0.92 (95% CI 0.87, 0.96, *p* < 0.001), respectively. We did not observe significant associations between serum ALT (HR _per 1-SD_ 0.99, 95% CI 0.94, 1.04, *p* = 0.62), GGT (HR _per 1-SD_ 1.02, 95% CI 0.97, 1.07, *p* = 0.43), and TP (HR _per 1-SD_ 0.97, 95% CI 0.93, 1.01, *p* = 0.16) and IBD risk. Moreover, recalibration of intraclass correlation coefficients led to stronger associations between these biomarkers and IBD (Table 3).

**Table 3:** Associations between serum liver function biomarkers in quintiles and risk of IBD

	Quintile 1	Quintile 2	Quintile 3	Quintile 4	Quintile 5	Per 1-SD increment	*P*-nonlinearity
Albumin							0.002
Median (IQR)	42.0 (41.0, 42.6)	43.9 (43.5, 44.2)	45.2 (44.9, 45.5)	46.5 (46.2, 46.9)	48.5 (47.9, 49.5)		
Cases/person-years	598/977,731	492/981,054	400/988,196	384/986,437	354/982,820		
HR (95% CI)^a^	Ref	0.81 (0.72, 0.92)	0.66 (0.58, 0.75)	0.63 (0.55, 0.72)	0.59 (0.51, 0.67)	0.83 (0.79, 0.86)	
HR (95% CI)^b^	Ref	0.87 (0.77, 0.98)	0.72 (0.64, 0.82)	0.71 (0.62, 0.81)	0.67 (0.58, 0.77)	0.86 (0.83, 0.90)	
HR (95% CI)^c^	Ref	0.74 (0.57, 0.96)	0.49 (0.38, 0.65)	0.47 (0.35, 0.63)	0.42 (0.31, 0.57)	0.72 (0.67, 0.80)	
Alkaline phosphatase							0.58
Median (IQR)	56.7 (51.1, 60.7)	69.7 (67.0, 72.3)	80.0 (77.4, 82.6)	91.5 (88.3, 95.1)	111.6 (104.5, 123.1)		
Cases/person-years	344/994,907	370/990,131	391/984,979	522/976,839	601/969,382		
HR (95% CI)^a^	Ref	1.05 (0.91, 1.22)	1.12 (0.96, 1.29)	1.51 (1.32, 1.73)	1.78 (1.56, 2.04)	1.25 (1.20, 1.31)	
HR (95% CI)^b^	Ref	1.02 (0.88, 1.18)	1.05 (0.91, 1.21)	1.37 (1.19, 1.57)	1.49 (1.30, 1.71)	1.18 (1.13, 1.24)	
HR (95% CI)^c^	Ref	1.03 (0.85, 1.24)	1.07 (0.88, 1.28)	1.51 (1.25, 1.80)	1.68 (1.41, 2.01)	1.24 (1.17, 1.32)	
Alanine transaminase							0.76
Median (IQR)	12.3 (10.7, 13.4)	16.2 (15.3, 17.0)	19.9 (18.9, 20.9)	24.9 (23.4, 26.7)	35.9 (31.7, 43.3)		
Cases/person-years	404/985,739	466/982,314	423/983,661	463/980,748	472/983,776		
HR (95% CI)^a^	Ref	1.10 (0.96, 1.26)	0.96 (0.84, 1.11)	1.04 (0.90, 1.19)	1.05 (0.91, 1.21)	1.01 (0.96, 1.06)	
HR (95% CI)^b^	Ref	1.12 (0.98, 1.28)	0.96 (0.84, 1.11)	1.02 (0.88, 1.17)	1.00 (0.86, 1.15)	0.99 (0.94, 1.04)	
HR (95% CI)^c^	Ref	1.23 (0.96, 1.57)	0.93 (0.73, 1.21)	1.04 (0.79, 1.33)	1.00 (0.76, 1.29)	0.98 (0.89, 1.07)	
Aspartate transaminase							0.29
Median (IQR)	18.3 (17.0, 19.3)	21.6 (20.9, 22.2)	24.2 (23.6, 24.9)	27.4 (26.5, 28.5)	33.7 (31.4, 37.8)		
Cases/person-years	464/991,971	444/1,002,541	457/961,705	447/996,168	416/963,853		
HR (95% CI)^a^	Ref	0.90 (0.79, 1.02)	0.93 (0.82, 1.06)	0.86 (0.75, 0.98)	0.81 (0.71, 0.93)	0.94 (0.90, 0.98)	
HR (95% CI)^b^	Ref	0.94 (0.82, 1.07)	0.99 (0.87, 1.13)	0.92 (0.80, 1.05)	0.85 (0.74, 0.97)	0.95 (0.91, 0.99)	
HR (95% CI)^c^	Ref	0.90 (0.70, 1.13)	0.98 (0.78, 1.24)	0.86 (0.67, 1.09)	0.75 (0.58, 0.95)	0.91 (0.85, 0.98)	
Gamma-glutamyl transferase							0.56
Median (IQR)	14.5 (12.7, 15.8)	19.8 (18.5, 21.2)	26.0 (24.2, 27.9)	35.7 (32.6, 39.5)	61.1 (51.0, 80.3)		
Cases/person-years	393/997,279	383/995,403	449/971,545	473/982,310	530/969,701		
HR (95% CI)^a^	Ref	0.92 (0.80, 1.06)	1.08 (0.94, 1.24)	1.10 (0.96, 1.27)	1.25 (1.09, 1.43)	1.10 (1.04, 1.15)	
HR (95% CI)^b^	Ref	0.88 (0.76, 1.01)	0.98 (0.85, 1.12)	0.95 (0.83, 1.10)	1.02 (0.88, 1.18)	1.02 (0.97, 1.07)	
HR (95% CI)^c^	Ref	0.84 (0.69, 1.01)	0.97 (0.80, 1.17)	0.93 (0.77, 1.14)	1.03 (0.84, 1.25)	1.03 (0.96, 1.10)	
Total bilirubin							< 0.001
Median (IQR)	5.3 (4.8, 5.7)	6.7 (6.4, 7.0)	8.0 (7.7, 8.4)	9.7 (9.2, 10.3)	13.6 (12.1, 16.7)		
Cases/person-years	548/984,901	442/980,557	419/985,883	431/984,052	388/980,845		
HR (95% CI)^a^	Ref	0.77 (0.68, 0.88)	0.70 (0.62, 0.80)	0.70 (0.62, 0.80)	0.62 (0.54, 0.71)	0.87 (0.83, 0.91)	
HR (95% CI)^b^	Ref	0.83 (0.73, 0.94)	0.78 (0.69, 0.89)	0.80 (0.70, 0.91)	0.73 (0.63, 0.83)	0.92 (0.87, 0.96)	
HR (95% CI)^c^	Ref	0.77 (0.64, 0.92)	0.70 (0.59, 0.85)	0.73 (0.61, 0.88)	0.64 (0.52, 0.77)	0.89 (0.82, 0.94)	
Total protein							0.011
Median (IQR)	67.5 (66.2, 68.4)	70.3 (69.7, 70.8)	72.3 (71.8, 72.8)	74.4 (73.8, 75.0)	77.7 (76.6, 79.4)		
Cases/person-years	478/987,545	421/990,675	475/984,922	401/980,838	453/972,258		
HR (95% CI)^a^	Ref	0.88 (0.77, 1.00)	1.00 (0.88, 1.14)	0.85 (0.75, 0.97)	0.97 (0.85, 1.10)	0.97 (0.93, 1.02)	
HR (95% CI)^b^	Ref	0.89 (0.78, 1.01)	1.01 (0.89, 1.14)	0.85 (0.75, 0.97)	0.96 (0.84, 1.09)	0.97 (0.93, 1.01)	
HR (95% CI)^c^	Ref	0.78 (0.59, 1.02)	1.02 (0.78, 1.32)	0.71 (0.54, 0.94)	0.92 (0.69, 1.20)	0.94 (0.86, 1.02)	

*BMI* body mass index, *CI* confidence interval, *HR* hazard ratio, *IBD* inflammatory bowel disease, *IQR* interquartile range, *SD* standard deviation

^a^Model 1 was adjusted for sex, age and ethnicity

^b^Model 2 was further adjusted for BMI, smoking status, physical activity level, alcohol consumption, Townsend deprivation index, C-reactive protein, healthy diet and Charlson Comorbidity Index based on Model 1

^c^Recalibrated multivariable estimates accounting for dilution bias using the intraclass correlation coefficients calculated in the subsample of participants with repeat measurements of circulating liver function markers

**Fig. 1:**
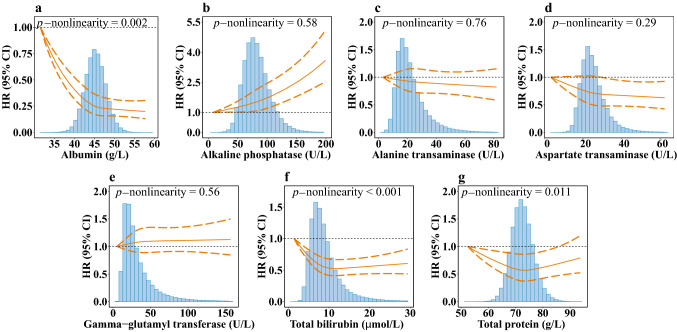
Nonlinear associations between serum liver function biomarkers and risk of IBD. **a** albumin; **b** alkaline phosphatase; **c** alanine transaminase; **d** aspartate transaminase; **e** gamma-glutamyl transferase; **f** total bilirubin; **g** total protein. The reference levels were set as the lowest values of each exposure, respectively. The vertical axis represents the risk of IBD based on the fully adjusted model. The solid line in orange represents hazard ratios, and dashed lines in orange represent 95% confidence intervals. Density plots of each biomarker were presented in gray. *CI* confidence interval; *HR* hazard ratio; *IBD* inflammatory bowel disease

Results from RCS analysis showed a statistically significant nonlinearity for ALB, TB and TP with incident IBD (*p*-nonlinearity = 0.002, < 0.001, 0.011, respectively). For serum ALB, the RCS curves showed that the HR decreased from 1 to 0.25 (95% CI 0.17, 0.36) rapidly when the concentrations increased from the lowest levels of 31.5 to 45 g/L. Serum TB and TP at the concentrations of 11 μmol/L and 73 g/L showed the smallest HR of 0.52 (95% CI 0.41, 0.67) and 0.56 (95% CI 0.37, 0.86) in RCS curves, respectively (Fig. 1).

**Fig. 2:**
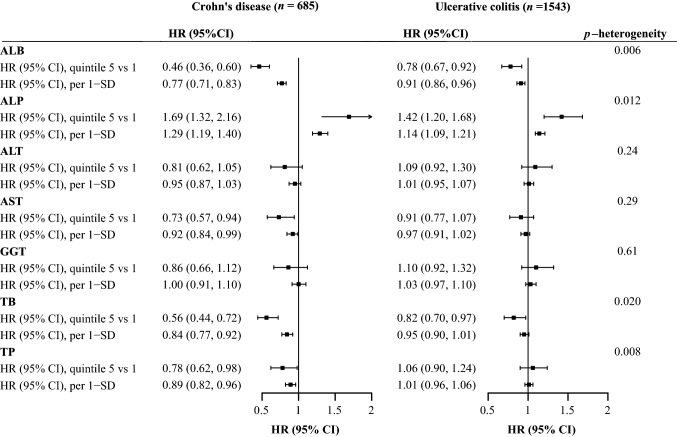
Forest plot for associations between serum liver function biomarkers and risk of Crohn's disease and ulcerative colitis. Hazard ratios with 95% confidence intervals were calculated based on the fully adjusted model

As shown in Fig. 2**,** when investigating the incidence of CD and UC independently, we found stronger associations of ALB, ALP, TB, TP with incident CD than UC (all *p*-heterogeneity < 0.05). For each 1-SD increment, the HRs (95% CIs) for associations of ALB, ALP, TB, TP with incident CD were 0.77 (95% CI 0.71, 0.83, *p* < 0.001), 1.29 (95% CI 1.19, 1.40, *p* < 0.001), 0.84 (95% CI 0.77, 0.92, *p* < 0.001), 0.89 (95% CI 0.82, 0.96, *p* = 0.002), respectively, while the HRs (95% CIs) for associations of ALB, ALP, TB, TP with incident UC were 0.91 (95% CI 0.86, 0.96, *p* < 0.001), 1.14 (95% CI 1.09, 1.21, *p* < 0.001), 0.95 (95% CI 0.90, 1.01, *p* = 0.080), 1.01 (95% CI 0.96, 1.06, *p* = 0.67), respectively. When we further explored the subtypes of CD and UC according to disease extent, we found significant heterogeneity in the associations of AST and TP across disease extent of CD (both *p*-heterogeneity < 0.05, Table S6). In contrast, the associations of ALP differed across disease extent of UC (*p*-heterogeneity = 0.015, Table S7). We observed a significant association between GGT and CD with perianal disease (HR per 1-SD 1.55, 95% CI 1.04, 2.31, *p* = 0.032), but not other liver biomarkers.

### Subgroup and sensitivity analysis

Subgroup analyses (Table S8) found that the associations between MAFLD and IBD were stronger (*p*-interaction = 0.045) in females (HR _per 1-SD_ 1.21, 95% CI 1.06, 1.38, *p* = 0.004) than males (HR _per 1-SD_ 1.05, 95% CI 0.93, 1.18, *p* = 0.46). The associations between MAFLD and IBD were not altered when stratified by age, alcohol consumption, smoking status, and BMI (All *p*-interaction > 0.10). Regarding associations between serum liver function biomarkers and incident IBD (Table S8), the inverse association of ALB with subsequent IBD was stronger in individuals aged below 60 at recruitment (HR _per 1-SD_ 0.83 vs. 0.91, *p*-interaction = 0.026). Furthermore, the association between TP and incident IBD was strengthened in females (HR _per 1-SD_ 0.88 vs. 0.96, *p*-interaction = 0.005) and previous or current smokers (HR _per 1-SD_ 0.86 vs. 1.00, *p*-interaction < 0.001).

The association between MAFLD and incident IBD remained consistent after excluding incident IBD in the first 2-year period (HR 1.13, 95% CI 1.03, 1.24, *p* = 0.008), baseline CRC (HR 1.12, 95% CI 1.02, 1.22, *p* = 0.016), other liver diseases (HR 1.12, 95% CI 1.02, 1.22, *p* = 0.017), refilling the missing values using multiple imputations (HR 1.11, 95% CI 1.01, 1.21, *p* = 0.032), excluding new-onset MAFLD identified in the repeat assessment (HR 1.11, 95% CI 1.01, 1.21, *p* = 0.030), adjusting for use of non-steroidal anti-inflammatory drugs (HR 1.10, 95% CI 1.01, 1.20, *p* = 0.036), antibiotics (HR 1.12, 95% CI 1.02, 1.22, *p* = 0.015), or proton-pump inhibitors (HR 1.10, 95% CI 1.00, 1.20, *p* = 0.043) as shown in Table S9. The associations between liver function biomarkers and incident IBD remained significant during the sensitivity analysis. As shown in Table S10, we did not observe significant association between AST to ALT ratio and IBD (HR _per 1-SD_ 1.00, 95% CI 0.95, 1.04, *p* = 0.85). As shown in Table S11, though level of AST (HR _per 1-SD_ 0.94, 95% CI 0.95, 1.04, *p* = 0.036) and TB (HR _per 1-SD_ 0.88, 95% CI 0.82, 0.94, *p* < 0.001) was shown significant inverse associations with IBD among individuals with AST and TB within normal range, borderline positive associations were observed for individual with AST (HR _per 1-SD_ 1.25, 95% CI 0.99, 1.59, *p* = 0.063) and TB (HR _per 1-SD_ 1.25, 95% CI 0.98, 1.58, *p* = 0.070) above normal range. In Table S12, using BAAT score evaluating the risk of advanced liver fibrosis, we found that compared with participants without MAFLD, participants with MAFLD with high risk of advanced liver fibrosis were associated with increased risk of IBD (HR 1.12, 95% CI 1.02, 1.23, *p* = 0.013), especially for CD (HR 1.32, 95% CI 1.11, 1.55, *p* = 0.001), but not UC (HR 1.05, 95% CI 0.94, 1.17, *p* = 0.40). Individuals with MAFLD without a high risk of advanced fibrosis also were significantly associated with incident CD compared with those without MAFLD (HR 1.58, 95% CI 1.12, 1.22, *p* = 0.008).

## Discussion

In this large prospective cohort study, we followed 405,413 individuals to evaluate the association between MAFLD, circulating liver function markers, and incident IBD risk. The primary analysis found that individuals with MAFLD had a higher risk of incident IBD than those without MAFLD. As for the subtypes of IBD, the association between MAFLD and CD incidence was consistent and significant, while no significant association was observed between MAFLD and UC incidence. During the secondary analysis, we found that the low circulating levels of ALB and high circulating levels of ALP were associated with increased risk of IBD. Ranging within normal range, serum AST and TB were inversely related to IBD. However, for serum AST and TB ranging above the normal ranges, borderline positive relation to IBD was detected. The present study provides compelling evidence showing that individuals with MAFLD would higher risk of developing IBD, especially CD. The secondary analysis of surrogate markers of liver function indicated the association between liver dysfunction and IBD.

Our data substantiated that the risk of incident IBD is higher in individuals with MAFLD than in the general population. Although the link between MAFLD and IBD has been largely understudied, current clinical guidelines for the management of MAFLD and a recent cohort study both reported that manifestations of MAFLD extend beyond the hepatobiliary system and might have significant interplay with the gastrointestinal tract [11, 36]. A systematic review, aiming to interpret the relationship between liver disease and IBD, indicated that gut microbiota might be altered in individuals with chronic liver disease, especially small intestinal bacterial overgrowth, leading to increased intestinal permeability and increased burden of incident IBD, which corroborated our findings. In this study, individuals with MAFLD have higher incident CD risks than incident UC risks. A potential explanation is that individuals with CD are more likely to have small bowel inflammation, which would be affected by the disruption of the bile acid metabolism in the ileum because of MAFLD [37].

We demonstrated MAFLD was associated with 150% increased risk of CD with perianal disease. Since there are only 37 cases of CD with perianal disease identified in this study, relevant clinical data and further studies on exploring underlying mechanism of the association between MAFLD and CD with perianal disease are needed. Individuals with MAFLD with high risk of advanced fibrosis were associated with development of IBD. This finding revealed that the severity of MAFLD may affect the development of IBD. But for CD, MAFLD with and without high risk of fibrosis is both associated with increased risk of CD, and there is no difference between their estimates. This may suggest that the severity of liver fibrosis does not influence the association between MAFLD and CD. As indicated by current review, there have not been any standard non-invasive algorithms in approach to liver fibrosis and the results of assessments of existing non-invasive scores are not consistent among existing evidence [38]. Therefore, these exploratory findings can serve as the start point for future studies on the severity of liver fibrosis and development of IBD and can be better clarified with liver biopsy data.

Our results showed that a 1-SD increase within the normal ranges of ALB, AST, and TB was associated with a 5% to 14% reduced risk of IBD. No clinical significance has yet been recognized for individuals with relatively low levels of liver enzymes and TB within or below the normal ranges. However, a systematic review by Kunutsor et al. showed that lower circulating liver function biomarker levels might reflect reduced liver function in the general population [39]. In addition, it is widely acknowledged that abnormally elevated levels of AST and TB reflect impaired liver function [19]. Our findings demonstrated borderline positive associations with IBD risk, when serum AST and TB increased above the upper limits. Our findings implied that decreased liver function, associated with lower levels of circulating liver function markers within the normal ranges, could be associated with higher IBD risk. Interestingly, we also detected a positive association between ALP levels and the risk of incident IBD. In line with our findings, previous clinical reports and laboratory studies detected increased serum ALP concentrations in the presence of vasculitis [40]. Increased ALP activity was strongly associated with the influx of inflammatory cells, which may accelerate IBD development [41]. Taken together, we found that the circulating levels of liver dysfunction biomarkers were surrogate indicators for the increased risk of IBD incidence.

We demonstrated that the relationship between TB and IBD is predominant in CD, and the associations of AST and TP are stronger in colonic CD. This finding is broadly consistent with previous evidence that the liver-microbiota-gut axis may be of particular importance to the pathological changes in the colon [42]. An increasing body of evidence suggests that high concentrations of liver-derived metabolites in the colon due to enterohepatic cycling are associated with the colon’s higher microbial abundance [43, 44]. The correlation between decreased ALB levels and increased risk of incident IBD was stronger in participants of younger age, and this finding could be related to the accumulation of the metabolic risk factors with aging [45]. Consistently, a previous study indicated an inverse association between TP (composed of ALB and globulin) and IBD risk, which was probably attributable to ALB [46].

Strengths of our study include the large sample size, prospective design, long-term follow-up, and comprehensive measurements of blood-related biomarkers in the UK Biobank that enabled the identification of MAFLD and evaluation of the association between MAFLD and risk of incident IBD and dose–response relationship of various liver function markers with incident IBD and its subtypes. The UK Biobank database also allowed us to assess the reproducibility and correct for dilution bias using the method developed to address regression dilution [42]. We excluded individuals with incident IBD in the first year during follow-up, avoiding the confounding effect of delay in IBD diagnosis in the real world, and adjusted the multivariable models by socioeconomic and lifestyle indicators. Besides, we excluded incident IBD in the first two years of follow-up during the sensitivity analysis, reducing potential confounding effects from reverse causality. Those sensitivity analyses support the robustness of the study findings.

However, with any observational study, the possibility of residual confounding bias cannot be ruled out despite adjusting for numerous health-related factors. Other limitations of this study should also be noted. Firstly, the standard diagnosis of fatty liver disease should have been based on imaging or biopsy. Due to the lack of baseline imaging data, fatty liver was identified by serum biomarkers. This limitation may affect the accuracy of diagnosis of fatty liver disease for included individuals. However, fatty liver index has shown good performance in detecting fatty liver in several population studies [47]. Another population-based cohort in the UK showed that the accuracy of diagnoses of fatty liver disease using fatty liver index ≥ 60 and ultrasound liver fat score was concordant (kappa = 0.79) [48]. Secondly, biomarkers of liver function were collected at baseline, and repeated biomarker assessments were only available for a small subset of participants, which could not fully represent the changes alongside the development of individuals' health and disease status. Thirdly, although the ethical and socioeconomic background of individuals in the UK biobank was varied, participants of these studies were predominantly non-Hispanic white, which may limit the generalizability and transferability of the results. Last and most importantly, due to the nature of the observational study, causality could not be determined in this study.

## Conclusions

In summary, in this large prospective cohort study, individuals with MAFLD have a higher risk of incident IBD, especially for subsequent CD, but not UC. Circulating levels of liver function biomarkers as the surrogate indicators of MAFLD were also associated with IBD risk. Our study provides preliminary clinical evidence indicating that individuals with MAFLD should be paid additional attention for the prevention of IBD incidence. Multi-centered prospective cohort studies are needed to verify our findings. Further studies are warranted to assess the effect of current MAFLD treatment and the severity of MAFLD on IBD development.

## Supplementary Information

Below is the link to the electronic supplementary material.

Supplementary file 1 (DOCX 121 KB)

## Data Availability

The datasets analyzed during the current study are available in a public, open-access repository (https://www.ukbiobank.ac.uk/).

## Abbreviations

ALB: Albumin
ALP: Alkaline phosphatase
ALT: Alanine transaminase
AST: Aspartate transaminase
BMI: Body mass index
CCI: Charlson Comorbidity Index
CD: Crohn’s disease
CI: Confidence interval
GGT: Gamma-glutamyl transferase
HR: Hazard ratio
IBD: Inflammatory bowel disease
IQR: Interquartile range
MAFLD: Metabolic dysfunction-associated fatty liver disease
RCS: Restricted cubic spline
SD: Standard deviation
TB: Total bilirubin
TP: Total protein
UC: Ulcerative colitis
