# Supplementary material for: Metabolic dysfunction-associated fatty liver disease and liver function markers are associated with Crohn’s disease but not Ulcerative Colitis: a prospective cohort study
Source: Hepatol Int. 2022 Oct 4;17(1):202–14. doi: 10.1007/s12072-022-10424-6 (PMC9895026; doi:10.1007/s12072-022-10424-6)
Supplement: Supplementary file 1 — (DOCX 121 KB) [file 12072_2022_10424_MOESM1_ESM.docx]

SUPPLEMENTARY MATERIALS

Metabolic dysfunction-associated fatty liver disease and liver function markers are associated with Crohn’s disease but not Ulcerative Colitis: a prospective cohort study

Jie Chen, Lintao Dan, Xinru Tu, Yuhao Sun, Minzi Deng, Xuejie Chen, Therese Hesketh, Ran Li, Xiaoyan Wang, Xue Li

[Supplementary figure 2](#_Toc112350011)

[Fig. S1 Flow chart of the inclusion of the participants in the UK Biobank 2](#_Toc112350012)

[Supplementary Tables 3](#_Toc112350013)

[Table S1. Definition of metabolic dysfunction-associated fatty liver disease 3](#_Toc112350014)

[Table S2. ICD codes for diagnosis of IBD and its phenotype 5](#_Toc112350015)

[Table S3. Percentage of participants within a normal range of serum liver function biomarkers 6](#_Toc112350016)

[Table S4. Age-adjusted correlation coefficients 7](#_Toc112350017)

[Table S5. Intraclass correlation coefficients of circulating liver function markers between two measurements 8](#_Toc112350018)

[Table S6. Associations between serum liver function biomarkers and risk of different anatomic distributions of CD 9](#_Toc112350019)

[Table S7. Associations between serum liver function biomarkers and different anatomic distributions of UC 10](#_Toc112350020)

[Table S8. Subgroup analyses for associations of MALFD and serum liver function biomarkers (per 1-SD increment) with risk of IBD ^a^ 12](#_Toc112350021)

[Table S9. Sensitivity analyses for associations of MAFLD and serum liver function biomarkers with risk of IBD 14](#_Toc112350022)

[Table S10. Association between aspartate transaminase to alanine transaminase ratio in quintiles and risk of IBD 16](#_Toc112350023)

[Table S11. Association between liver function biomarkers (in per SD increment) and risk of IBD stratified by whether the upper limit of the normal concentration range is exceeded 17](#_Toc112350024)

[Table S12 Association between MAFLD with different risk of advanced fibrosis and risk of IBD, CD and UC according to BAAT score 18](#_Toc112350025)

[Supplementary Methods 19](#_Toc112350026)

# Supplementary figure

**Participants who enrolled in the UK Biobank (*n* = 502,460)**

**Participants excluded:**

1**.** Participants without available information of fatty liver index and other information to identify prevalence of MAFLD (*n*=50,144)

2**.** Participants without available information of liver circulation biomarkers (*n*=22,064)

3. participants with baseline IBD (*n*=5537)

4. participants who were indicated as outliers (*n*=19,163)

5. IBD cases that occurred in the first 1–year follow up (*n*=139)

**Participants in analysis**

**(*n* = 405,413)**

## Fig. S1 Flow chart of the inclusion of the participants in the UK Biobank

# Supplementary Tables

## Table S1. Definition of metabolic dysfunction-associated fatty liver disease

| Definition items | Corresponding items in the UK Biobank | | Description |
| --- | --- | --- | --- |
| **Hepatic steatosis** | Fatty liver index ≥60 [1] | (e^0.953*ln (TG) + 0.139*BMI + 0.718*ln (GGT) + 0.053*WC - 15.745^) / (1 + e^0.953*ln (TG) + 0.139*BMI + 0.718*ln(GGT) + 0.053*WC - 15.745^) ×100 | The FLI is a simple and commonly accepted indicator for human fatty liver in the absence of imaging and histological data [2] |
| **Met one of three** |  |  |  |
| **Overweight or obesity** | BMI≥25 kg/m^2^ | | Based on an international expert consensus statement [3] |
| **Type 2 diabetes mellitus** | Met one of three | Glycated hemoglobin A1c > 47 mmol/mol | Based on an international expert consensus statement [3] |
|  |  | ICD-10 codes of E11.0-E11.9 or ICD-9 code of 250 | Based on an international expert consensus statement [3] |
|  |  | Regularly took medication for diabetes | Metformin: metformin, rosiglitazone + metformin  Sulphonylureas: glipizide, glipizide product, glibenclamide, gliclazide, glimepiride Meglitinides: starlix, nateglinide, repaglinide Glitazones: troglitazone (none report), pioglitazone, rosiglitazone,  acarbose [4, 5] |
| **Metabolic abnormalities** |  |  |  |
| At least two | Increased waist circumference | ≥ 102 cm for men and ≥88 cm for women | Based on an international expert consensus statement [3] |
|  | Arterial hypertension | systolic ≥130 or diastolic ≥85 mmHg | Based on an international expert consensus statement [3] |
|  |  | use of antihypertensive medication | Based on an international expert consensus statement [3] |
|  | Hypertriglyceridemia | ≥ 1.7 mmol/L | Based on an international expert consensus statement [3] |
|  |  | use of a relevant drug | Fibrates: bezafibrate, clofibrate, fenofibrate, ciprofibrate, gemfibrozil, gemfibrozil product, bezafibrate product Niacin: nicotinic acid product, Niacin [6] |
|  | Low HDL cholesterol | 1.0mmol/L for men and < 1.3 mmol/L for women | Based on an international expert consensus statement [3] |
|  |  | Use of a relevant drug | Niacin: nicotinic acid product, Niacin gemfibrozil, gemfibrozil product, fenofibrate [6] |
|  | Prediabetes | Glycated hemoglobin A1c 39-47 mmol/mol | Based on an international expert consensus statement [3] |
|  | Insulin resistance |  | Not applicable in the UK biobank [7] |
|  | Subclinical inflammation | C-reactive protein > 2mg/L | Based on an international expert consensus statement s [3] |

Abbreviations: BMI, body biomass index; GGT, gamma-glutamyl transferase; TG, triglycerides; WC, waist circumstances

## Table S2. ICD codes for diagnosis of IBD and its phenotype

| Diagnosis | ICD-10 | ICD-9 |
| --- | --- | --- |
| IBD | K50, K50.0, K50.1, K50.8, K50.9, K51, K51.0, K51.1, K51.2, K51.3, K51.4, K51.5, K51.8, K51.9 | 555, 5550, 5551, 5552, 5559, 556, 5569 |
| **Subtypes** |  |  |
| Crohn's disease | K50, K50.0, K50.1, K50.8, K50.9 | 555, 5550, 5551, 5552, 5559, 556, 5569 |
| Ulcerative colitis | K51, K51.0, K51.1, K51.2, K51.3, K51.4, K51.5, K51.8, K51.9 | 556, 5569 |
| **Disease extent ^a^** |  |  |
| Crohn's disease |  |  |
| Ileal CD | K50.0 | 5550 |
| Colonic CD | K50.1 | 5551 |
| Ileocolonic or unspecified CD | K50.8, K50.9 | 555, 5552, 5559 |
| Ulcerative colitis |  |  |
| Ulcerative proctitis | K51.2 |  |
| Left sided UC | K51.3, K51.5 |  |
| Extensive UC | K51.0, K51.1 |  |
| Unspecified UC | K51.4, K51.8, K51.9 | 556, 5569 |
| **CD with** **perianal disease** | Diagnosis with CD and ICD-10 code K603, K604, K605, K610, K611, K612, K613, K614, K624 OR operation code (OPCS-4) H564, H562, H532, H548, H549, H581, H582, H583, H444, H551, H552, H553, H554, H556, H557, H558, H559, H414, P134, P253 | Diagnosis with CD and ICD-9 code 5651, 5569 OR operation code (OPCS-4) |

^a^ Disease extent was defined by the highest available degree ever of anatomic distribution of inflammation according to International Disease Classification code [8].

Abbreviations: IBD, inflammatory bowel disease; ICD, international classification disease.

## Table S3. Percentage of participants within a normal range of serum liver function biomarkers

| Biomarker | Normal range ^a^ | Percentage of participants within the normal range |
| --- | --- | --- |
| Albumin | 35-57 g/L | 99.98% |
| Alkaline phosphatase | 34-104 U/L | 86% |
| Alanine transaminase | 7-52 U/L | 97% |
| Aspartate transaminase | 13-39 U/L | 96% |
| Gamma-glutamyl transferase | 9-64 U/L | 91% |
| Total bilirubin | 5-17 μmol/L | 88% |
| Total protein | 64-89 g/L | 99% |

^a^ The normal range was based on the Beckman Coulter AU5800 determined reference range, which was the analytical platform for serum liver function biomarkers in the UK Biobank (<https://www.beckmancoulter.com/support/tech-docs>)

## Table S4. Age-adjusted correlation coefficients ^a, b^

|  | ALB | ALP | ALT | AST | BMI | GGT | TB | TG | TP | WC | Age |
| --- | --- | --- | --- | --- | --- | --- | --- | --- | --- | --- | --- |
| ALB | 1 | -0.005 | 0.15 | 0.17 | -0.12 | 0.13 | 0.21 | 0.045 | 0.47 | -0.056 | -0.14 |
| ALP | -0.005 | 1 | 0.16 | 0.11 | 0.16 | 0.19 | -0.12 | 0.15 | 0.12 | 0.12 | 0.18 |
| ALT | 0.15 | 0.16 | 1 | 0.69 | 0.33 | 0.58 | 0.12 | 0.33 | 0.13 | 0.42 | 0.025 |
| AST | 0.17 | 0.11 | 0.69 | 1 | 0.11 | 0.39 | 0.16 | 0.14 | 0.19 | 0.19 | 0.12 |
| BMI | -0.12 | 0.16 | 0.33 | 0.11 | 1 | 0.32 | -0.064 | 0.36 | 0.034 | 0.80 | 0.066 |
| GGT | 0.13 | 0.19 | 0.58 | 0.39 | 0.32 | 1 | 0.092 | 0.34 | 0.16 | 0.43 | 0.11 |
| TB | 0.21 | -0.12 | 0.12 | 0.16 | -0.064 | 0.092 | 1 | 0.11 | 0.079 | 0.057 | 0.006 |
| TG | 0.045 | 0.15 | 0.33 | 0.14 | 0.36 | 0.34 | 0.11 | 1 | 0.051 | 0.42 | 0.12 |
| TP | 0.47 | 0.12 | 0.13 | 0.19 | 0.034 | 0.16 | 0.079 | 0.051 | 1 | 0.037 | -0.068 |
| WC | -0.056 | 0.12 | 0.42 | 0.19 | 0.80 | 0.43 | 0.057 | 0.42 | 0.037 | 1 | 0.12 |
| Age | -0.14 | 0.18 | 0.025 | 0.12 | 0.066 | 0.11 | 0.006 | 0.12 | -0.068 | 0.12 | 1 |

^a^ All correlation coefficients are age adjusted using partial correlation except for those with age itself. Spearman correlation coefficient was calculated between age and other vicariate.

^b^ All *P* values < 0.01.

Abbreviations: ALB, albumin; ALP, alkaline phosphatase; ALT, alanine transaminase; AST, aspartate transaminase; BMI, body mass index; GGT, gamma-glutamyl transferase; TB, total bilirubin; TG, triglyceride; TP, total protein; WC, waist circumstances.

## Table S5. Intraclass correlation coefficients of circulating liver function markers between two measurements ^a^

|  | Intraclass correlation coefficients (95% CI) |
| --- | --- |
| Albumin | 0.46 (0.43, 0.49) |
| Alkaline phosphatase | 0.77 (0.75, 0.78) |
| Alanine transaminase | 0.55 (0.54, 0.56) |
| Aspartate transaminase | 0.56 (0.55, 0.57) |
| Body mass index | 0.92 (0.92, 0.93) |
| Gamma-glutamyl transferase | 0.73 (0.72, 0.74) |
| Total bilirubin | 0.71 (0.70, 0.72) |
| Triglyceride | 0.59 (0.58, 0.60) |
| Total protein | 0.47 (0.40, 0.53) |
| Waist circumstances | 0.85 (0.83, 0.87) |

^a^ Intraclass correlation coefficients was calculated in the subgroup of participants with all information of repeated measurement (2012-2013) of serum liver function biomarkers available and did not diagnose with IBD during the period between the two measurements (n=12,080)

Abbreviations: CI confidence interval.

## Table S6. Associations between serum liver function biomarkers and risk of different anatomic distributions of CD ^a^

|  | **Disease extent of CD** | | | *P*-_heterogeneity_ ^b^ |
| --- | --- | --- | --- | --- |
|  | Ileal CD (*n* = 137) | Colonic CD (*n* = 74) | Ileocolonic or unspecific CD (*n* = 474) |  |
| **Albumin** |  |  |  | 0.52 |
| HR (95% CI), quintile 5 vs 1 | 0.42 (0.24, 0.74) | 0.47 (0.21, 1.06) | 0.48 (0.35, 0.65) |  |
| HR (95% CI), per 1-SD increment | 0.70 (0.58, 0.84) | 0.75 (0.59, 0.96) | 0.79 (0.71, 0.87) |  |
| **Alkaline phosphatase** |  |  |  | 0.13 |
| HR (95% CI), quintile 5 vs 1 | 2.23 (1.27, 3.93) | 2.06 (0.91, 4.66) | 1.51 (1.13, 2.02) |  |
| HR (95% CI), per 1-SD increment | 1.52 (1.26, 1.84) | 1.16 (0.91, 1.47) | 1.25 (1.13, 1.38) |  |
| **Alanine transaminase** |  |  |  | 0.74 |
| HR (95% CI), quintile 5 vs 1 | 0.65 (0.36, 1.19) | 1.38 (0.60, 3.21) | 0.79 (0.58, 1.08) |  |
| HR (95% CI), per 1-SD increment | 0.88 (0.72, 1.07) | 0.94 (0.72, 1.22) | 0.96 (0.87, 1.07) |  |
| **Aspartate transaminase** |  |  |  | 0.031 |
| HR (95% CI), quintile 5 vs 1 | 0.47 (0.26, 0.87) | 1.12 (0.56, 2.23) | 0.75 (0.56, 1.01) |  |
| HR (95% CI), per 1-SD increment | 0.74 (0.61, 0.90) | 1.10 (0.86, 1.41) | 0.94 (0.85, 1.04) |  |
| **Gamma-glutamyl transferase** |  |  |  | 0.80 |
| HR (95% CI), quintile 5 vs 1 | 0.80 (0.43, 1.49) | 0.99 (0.46, 2.09) | 0.86 (0.63, 1.17) |  |
| HR (95% CI), per 1-SD increment | 0.96 (0.78, 1.18) | 1.08 (0.81, 1.42) | 1.00 (0.90, 1.12) |  |
| **Total bilirubin** |  |  |  | 0.44 |
| HR (95% CI), quintile 5 vs 1 | 0.52 (0.29, 0.92) | 0.47 (0.20, 1.13) | 0.58 (0.44, 0.78) |  |
| HR (95% CI), per 1-SD increment | 0.81 (0.66, 0.99) | 0.99 (0.76, 1.29) | 0.83 (0.74, 0.92) |  |
| **Total protein** |  |  |  | 0.049 |
| HR (95% CI), quintile 5 vs 1 | 0.63 (0.38, 1.05) | 1.35 (0.64, 2.83) | 0.78 (0.59, 1.02) |  |
| HR (95% CI), per 1-SD increment | 0.79 (0.67, 0.94) | 1.13 (0.90, 1.43) | 0.88 (0.80, 0.96) |  |

^a^ adjusted for age, sex, ethnicity, BMI, smoking status, physical activity level, alcohol consumption, Townsend deprivation index, C-reactive protein, healthy diet and Charlson Comorbidity Index

^b^ P for heterogeneity was calculated using the contrast method using the contrast method based on a fully unconstrained approach.

Abbreviations: BMI, body mass index; CD, Crohn's disease; CI confidence interval; GGT; HR, hazard ratio; SD, standard deviation.

Table S7. Associations between serum liver function biomarkers and different anatomic distributions of UC ^a^

|  | **Disease extent of UC** | | | | *P*-heterogeneity ^b^ |
| --- | --- | --- | --- | --- | --- |
|  | Ulcerative proctitis (*n* = 147) | Left sided UC (*n* = 147) | Pancolitis (n = 96) | Unspecific UC (*n* = 1153) |  |
| **Albumin** |  |  |  |  | 0.47 |
| HR (95% CI), quintile 5 vs 1 | 0.91 (0.55, 1.51) | 0.76 (0.46, 1.28) | 0.45 (0.22, 0.91) | 0.80 (0.66, 0.97) |  |
| HR (95% CI), per 1-SD increment | 0.93 (0.79, 1.10) | 0.96 (0.81, 1.13) | 0.78 (0.63, 0.96) | 0.91 (0.86, 0.97) |  |
| **Alkaline phosphatase** | |  |  |  | 0.015 |
| HR (95% CI), quintile 5 vs 1 | 1.51 (0.92, 2.50) | 0.88 (0.55, 1.43) | 1.06 (0.56, 2.01) | 1.56 (1.28, 1.91) |  |
| HR (95% CI), per 1-SD increment | 1.10 (0.93, 1.30) | 0.96 (0.80, 1.13) | 0.92 (0.74, 1.13) | 1.20 (1.13, 1.28) |  |
| **Alanine transaminase** | |  |  |  | 0.11 |
| HR (95% CI), quintile 5 vs 1 | 0.76 (0.44, 1.31) | 0.70 (0.39, 1.24) | 0.94 (0.46, 1.91) | 1.23 (1.01, 1.51) |  |
| HR (95% CI), per 1-SD increment | 0.88 (0.73, 1.07) | 0.86 (0.71, 1.05) | 0.97 (0.77, 1.22) | 1.05 (0.98, 1.12) |  |
| **Aspartate transaminase** | |  |  |  | 0.52 |
| HR (95% CI), quintile 5 vs 1 | 0.75 (0.42, 1.34) | 0.74 (0.41, 1.33) | 0.74 (0.39, 1.42) | 0.96 (0.79, 1.16) |  |
| HR (95% CI), per 1-SD increment | 0.88 (0.73, 1.05) | 0.94 (0.79, 1.12) | 0.89 (0.71, 1.10) | 0.99 (0.93, 1.05) |  |
| **Gamma-glutamyl transferase** | |  |  |  | 0.30 |
| HR (95% CI), quintile 5 vs 1 | 1.02 (0.57, 1.80) | 1.06 (0.62, 1.82) | 1.12 (0.56, 2.21) | 1.12 (0.91, 1.38) |  |
| HR (95% CI), per 1-SD increment | 0.87 (0.71, 1.06) | 1.13 (0.93, 1.38) | 1.06 (0.83, 1.35) | 1.04 (0.97, 1.12) |  |
| **Total bilirubin** |  |  |  |  | 0.46 |
| HR (95% CI), quintile 5 vs 1 | 0.78 (0.45, 1.37) | 0.56 (0.31, 1.02) | 0.84 (0.43, 1.65) | 0.86 (0.71, 1.04) |  |
| HR (95% CI), per 1-SD increment | 0.93 (0.77, 1.12) | 0.82 (0.67, 0.99) | 0.95 (0.75, 1.21) | 0.97 (0.91, 1.04) |  |
| **Total protein** |  |  |  |  | 0.21 |
| HR (95% CI), quintile 5 vs 1 | 0.95 (0.57, 1.59) | 0.80 (0.47, 1.36) | 1.02 (0.56, 1.85) | 1.12 (0.93, 1.35) |  |
| HR (95% CI), per 1-SD increment | 1.00 (0.85, 1.18) | 0.90 (0.77, 1.06) | 0.89 (0.72, 1.09) | 1.04 (0.98, 1.10) |  |

^a^ adjusted for age, sex, ethnicity, BMI, smoking status, physical activity level, alcohol consumption, Townsend deprivation index, C-reactive protein, healthy diet and Charlson Comorbidity Index

^b^ P for heterogeneity was calculated using the contrast method using the contrast method based on a fully unconstrained approach.

Abbreviations: BMI, body mass index; CI confidence interval; HR, hazard ratio; SD, standard deviation; UC, ulcerative colitis.

## Table S8. Subgroup analyses for associations of MALFD and serum liver function biomarkers (per 1-SD increment) with risk of IBD ^a^

|  | IBD cases | MAFLD ^b^ | ALB | ALP | ALT | AST | GGT | TB | TP |
| --- | --- | --- | --- | --- | --- | --- | --- | --- | --- |
| **Age at recruitment** | |  |  |  |  |  |  |  |  |
| <60 | 1156 | 1.11 (0.98, 1.26) | 0.83 (0.78, 0.88) | 1.16 (1.09, 1.23) | 1.01 (0.95, 1.08) | 0.96 (0.90, 1.02) | 1.01 (0.94, 1.09) | 0.91 (0.85, 0.98) | 0.95 (0.89, 1.01) |
| ≥60 | 1072 | 1.12 (0.98, 1.27) | 0.91 (0.86, 0.97) | 1.20 (1.12, 1.28) | 0.97 (0.91, 1.04) | 0.93 (0.88, 1.00) | 1.02 (0.95, 1.10) | 0.92 (0.85, 0.98) | 1.00 (0.94, 1.06) |
| *P*-interaction | | 0.80 | 0.026 | 0.92 | 0.87 | 0.081 | 0.73 | 0.36 | 0.26 |
| **Sex** |  |  |  |  |  |  |  |  |  |
| Male | 1121 | 1.05 (0.93, 1.18) | 0.84 (0.79, 0.90) | 1.23 (1.15, 1.31) | 0.98 (0.91, 1.04) | 0.93 (0.87, 0.99) | 0.99 (0.92, 1.06) | 0.96 (0.89, 1.02) | 1.01 (0.95, 1.07) |
| Female ^c^ | 1107 | 1.21 (1.06, 1.38) | 0.88 (0.83, 0.94) | 1.15 (1.07, 1.22) | 1.00 (0.93, 1.07) | 0.97 (0.91, 1.03) | 1.05 (0.98, 1.13) | 0.88 (0.81, 0.94) | 0.94 (0.89, 1.00) |
| *P*-interaction | | 0.045 | 0.87 | 0.45 | 0.48 | 0.39 | 0.14 | 0.005 | 0.15 |
| **Alcohol consumption** | |  |  |  |  |  |  |  |  |
| None to moderate | 454 | 1.14 (0.94, 1.40) | 0.85 (0.77, 0.93) | 1.19 (1.08, 1.31) | 0.95 (0.85, 1.06) | 0.91 (0.83, 1.01) | 0.95 (0.84, 1.07) | 0.88 (0.80, 0.98) | 0.99 (0.90, 1.09) |
| Heavy | 1774 | 1.12 (1.01, 1.23) | 0.87 (0.83, 0.91) | 1.18 (1.12, 1.24) | 0.99 (0.94, 1.05) | 0.96 (0.91, 1.01) | 1.04 (0.98, 1.10) | 0.93 (0.88, 0.98) | 0.97 (0.92, 1.01) |
| *P*-interaction | | 0.74 | 0.79 | 0.42 | 0.67 | 0.63 | 0.44 | 0.30 | 0.87 |
| **Smoking status** | |  |  |  |  |  |  |  |  |
| Never smoked | 645 | 1.18 (1.03, 1.35) | 0.87 (0.81, 0.93) | 1.15 (1.07, 1.23) | 0.95 (0.88, 1.02) | 0.94 (0.87, 1.00) | 1.02 (0.94, 1.10) | 1.00 (0.93, 1.08) | 0.97 (0.91, 1.03) |
| Previous or current smokers | 1583 | 1.09 (0.97, 1.22) | 0.86 (0.81, 0.91) | 1.21 (1.14, 1.29) | 1.02 (0.95, 1.08) | 0.96 (0.90, 1.02) | 1.02 (0.95, 1.09) | 0.86 (0.80, 0.91) | 0.97 (0.92, 1.03) |
| *P*-interaction | | 0.41 | 0.81 | 0.70 | 0.78 | 0.89 | 0.21 | <0.001 | 0.87 |
| **BMI, kg/m^2^** | |  |  |  |  |  |  |  |  |
| <25 | 650 | 1.20 (0.76, 1.90) | 0.88 (0.81, 0.95) | 1.09 (1.01, 1.19) | 0.96 (0.87, 1.05) | 0.93 (0.86, 1.02) | 1.12 (1.02, 1.23) | 0.88 (0.80, 0.96) | 1.01 (0.93, 1.09) |
| ≥25 | 1579 | 1.09 (0.98, 1.22) | 0.86 (0.82, 0.90) | 1.22 (1.16, 1.29) | 1.00 (0.95, 1.06) | 0.96 (0.91, 1.01) | 0.99 (0.93, 1.05) | 0.93 (0.88, 0.99) | 0.95 (0.91, 1.00) |
| *P*-interaction | | 0.40 | 0.36 | 0.77 | 0.65 | 0.18 | 0.006 | 0.72 | 0.11 |

^a^ Based on fully adjusted model (age, sex, ethnicity, BMI, smoking status, physical activity level, alcohol consumption, Townsend deprivation index, C-reactive protein, healthy diet and Charlson Comorbidity Index) without adjustment for the corresponding items.

^b^ For MAFLD, the fully adjusted model did not include the variable BMI and C-reactive protein given its definition in this study.

c In subgroup analysis of females, hormonal replacement therapy and oral contraceptive pills were included as covariates for additional adjustment.

Abbreviations: ALB, albumin; ALP, alkaline phosphatase; ALT, alanine transaminase; AST, aspartate transaminase; BMI, body mass index; CI confidence interval; GGT, gamma-glutamyl transferase; HR, hazard ratio; MAFLD, metabolic dysfunction-associated fatty liver disease; IBD, inflammatory bowel disease; SD, standard deviation; TB, total bilirubin; TP, total protein.

## Table S9. Sensitivity analyses for associations of MAFLD and serum liver function biomarkers with risk of IBD

|  | Excluding IBD cases occurred during the first 2-year period | Excluding participants with baseline colorectal cancer | Excluding participants with baseline liver disease | Additionally adjusted for use of non-steroidal anti-inflammatory drugs | Additionally adjusted for use of antibiotics | Additionally adjusted for use of proton pump inhibitors | Refilling missing values using the multiple imputation method | Excluding new-onset individuals with MAFLD |
| --- | --- | --- | --- | --- | --- | --- | --- | --- |
| **MAFLD ^a^** |  |  |  |  |  |  |  |  |
| HR (95% CI), vs non-MAFLD | 1.13 (1.03, 1.24) | 1.12 (1.02, 1.22) | 1.12 (1.02, 1.22) | 1.10 (1.01, 1.20) | 1.10 (1.00, 1.20) | 1.10 (1.00, 1.20) | 1.11 (1.01, 1.21) | 1.11 (1.01, 1.21) |
| **Albumin ^b^** |  |  |  |  |  |  |  |  |
| HR (95% CI), quintile 5 vs 1 | 0.69 (0.60, 0.79) | 0.67 (0.59, 0.77) | 0.66 (0.58, 0.76) | 0.67 (0.58, 0.76) | 0.67 (0.58, 0.76) | 0.67 (0.58, 0.76) | 0.66 (0.58, 0.76) | 0.66 (0.58, 0.76) |
| HR (95% CI), per 1-SD increment | 0.87 (0.83, 0.91) | 0.87 (0.83, 0.90) | 0.86 (0.83, 0.90) | 0.86 (0.83, 0.90) | 0.86 (0.83, 0.90) | 0.86 (0.83, 0.90) | 0.86 (0.83, 0.90) | 0.87 (0.82, 0.90) |
| **Alkaline phosphatase ^b^** | |  |  |  |  |  |  |  |
| HR (95% CI), quintile 5 vs 1 | 1.43 (1.24, 1.65) | 1.50 (1.30, 1.72) | 1.51 (1.31, 1.73) | 1.50 (1.31, 1.72) | 1.50 (1.30, 1.72) | 1.50 (1.30, 1.72) | 1.54 (1.34, 1.78) | 1.50 (1.30, 1.72) |
| HR (95% CI), per 1-SD increment | 1.18 (1.12, 1.23) | 1.18 (1.13, 1.24) | 1.19 (1.14, 1.24) | 1.19 (1.13, 1.24) | 1.18 (1.13, 1.24) | 1.18 (1.13, 1.24) | 1.19 (1.14, 1.25) | 1.19 (1.14, 1.25) |
| **Alanine transaminase ^b^** | |  |  |  |  |  |  |  |
| HR (95% CI), quintile 5 vs 1 | 1.00 (0.86, 1.16) | 0.99 (0.85, 1.14) | 1.00 (0.87, 1.16) | 0.99 (0.86, 1.15) | 0.99 (0.86, 1.14) | 0.99 (0.86, 1.14) | 1.01 (0.87, 1.18) | 1.02 (0.88, 1.17) |
| HR (95% CI), per 1-SD increment | 0.99 (0.94, 1.04) | 0.99 (0.94, 1.04) | 0.99 (0.94, 1.04) | 0.99 (0.94, 1.04) | 0.99 (0.94, 1.03) | 0.99 (0.94, 1.03) | 0.99 (0.94, 1.04) | 0.98 (0.94, 1.03) |
| **Aspartate transaminase ^b^** | |  |  |  |  |  |  |  |
| HR (95% CI), quintile 5 vs 1 | 0.83 (0.72, 0.96) | 0.84 (0.73, 0.96) | 0.86 (0.75, 0.98) | 0.85 (0.74, 0.97) | 0.84 (0.74, 0.97) | 0.84 (0.74, 0.97) | 0.86 (0.75, 1.00) | 0.83 (0.72, 0.95) |
| HR (95% CI), per 1-SD increment | 0.95 (0.90, 0.99) | 0.95 (0.90, 0.99) | 0.95 (0.91, 1.00) | 0.95 (0.91, 0.99) | 0.95 (0.91, 0.99) | 0.95 (0.91, 0.99) | 0.96 (0.92, 1.01) | 0.95 (0.91, 1.00) |
| **Gamma-glutamyl transferase ^b^** | |  |  |  |  |  |  |  |
| HR (95% CI), quintile 5 vs 1 | 1.04 (0.89, 1.21) | 1.02 (0.88, 1.18) | 1.02 (0.88, 1.18) | 1.01 (0.87, 1.17) | 1.00 (0.86, 1.16) | 1.00 (0.86, 1.16) | 1.05 (0.90, 1.22) | 1.04 (0.90, 1.21) |
| HR (95% CI), per 1-SD increment | 1.02 (0.97, 1.08) | 1.02 (0.97, 1.07) | 1.02 (0.97, 1.07) | 1.02 (0.97, 1.07) | 1.01 (0.96, 1.07) | 1.01 (0.96, 1.07) | 1.02 (0.97, 1.08) | 1.01 (0.96, 1.07) |
| **Total bilirubin ^b^** |  |  |  |  |  |  |  |  |
| HR (95% CI), quintile 5 vs 1 | 0.73 (0.63, 0.84) | 0.73 (0.63, 0.83) | 0.72 (0.63, 0.83) | 0.73 (0.64, 0.84) | 0.73 (0.64, 0.84) | 0.73 (0.64, 0.84) | 0.73 (0.64, 0.85) | 0.72 (0.63, 0.83) |
| HR (95% CI), per 1-SD increment | 0.91 (0.87, 0.96) | 0.92 (0.87, 0.96) | 0.92 (0.87, 0.96) | 0.92 (0.87, 0.96) | 0.92 (0.87, 0.96) | 0.92 (0.87, 0.96) | 0.92 (0.88, 0.97) | 0.92 (0.87, 0.96) |
| **Total protein ^b^** |  |  |  |  |  |  |  |  |
| HR (95% CI), quintile 5 vs 1 | 0.92 (0.80, 1.06) | 0.95 (0.83, 1.08) | 0.96 (0.84, 1.09) | 0.96 (0.84, 1.09) | 0.96 (0.84, 1.09) | 0.96 (0.84, 1.09) | 1.00 (0.87, 1.14) | 0.97 (0.85, 1.10) |
| HR (95% CI), per 1-SD increment | 0.96 (0.92, 1.00) | 0.97 (0.93, 1.01) | 0.97 (0.93, 1.01) | 0.97 (0.93, 1.01) | 0.97 (0.93, 1.01) | 0.97 (0.93, 1.01) | 0.98 (0.94, 1.03) | 0.98 (0.93, 1.02) |

^a^ adjusted for age, sex, ethnicity, smoking status, physical activity level, alcohol consumption, Townsend deprivation index, healthy diet and Charlson Comorbidity Index

^b^ adjusted for age, sex, ethnicity, BMI, smoking status, physical activity level, alcohol consumption, Townsend deprivation index, C-reactive protein, healthy diet and Charlson Comorbidity Index

Abbreviations: BMI, body mass index; CI confidence interval; HR, hazard ratio; IBD, inflammatory bowel disease; SD, standard deviation.

## Table S10. Association between aspartate transaminase to alanine transaminase ratio in quintiles and risk of IBD

|  | Quintile 1 | Quintile 2 | Quintile 3 | Quintile 4 | Quintile 5 | Per 1-SD increment |
| --- | --- | --- | --- | --- | --- | --- |
| **Aspartate transaminase to alanine transaminase ratio** | | |  |  |  |  |
| Median (IQR) | 0.81 (0.72, 0.88) | 1.03 (0.98, 1.08) | 1.21 (1.16, 1.25) | 1.41 (1.35, 1.47) | 1.75 (1.63, 1.97) |  |
| Cases/person-years | 471/986,040 | 458/983,642 | 489/983,688 | 419/984,152 | 393/978,714 |  |
| HR (95% CI) ^a^ | Ref | 0.98 (0.86, 1.11) | 1.06 (0.93, 1.20) | 0.92 (0.80, 1.05) | 0.89 (0.77, 1.02) | 0.97 (0.92, 1.01) |
| HR (95% CI) ^b^ | Ref | 1.01 (0.89, 1.15) | 1.13 (0.99, 1.28) | 1.00 (0.87, 1.15) | 0.98 (0.85, 1.13) | 1.00 (0.95, 1.04) |

^a^ Model 1 was adjusted for sex, age and ethnicity.

^b^ Model 2 was further adjusted for BMI, smoking status, physical activity level, alcohol consumption, Townsend deprivation index, C-reactive protein, healthy diet and Charlson Comorbidity Index based on Model 1.

Abbreviations: BMI, body mass index; CI confidence interval; HR, hazard ratio; IBD, inflammatory bowel disease; IQR, interquartile range; SD, standard deviation.

## Table S11. Association between liver function biomarkers (in per SD increment) and risk of IBD stratified by whether the upper limit of the normal concentration range is exceeded

|  | IBD cases | HR (95% CI) ^a^ |
| --- | --- | --- |
| **Alkaline phosphatase** |  |  |
| ≤ 104 U/L (*n* = 343,061) | 1747 | 1.18 (1.09, 1.27) |
| > 104 U/L (*n* = 62,352) | 481 | 1.15 (1.03, 1.27) |
| **Alanine transaminase** |  |  |
| ≤ 52 U/L (*n* = 396,068) | 2186 | 1.06 (0.70, 1.61) |
| > 52 U/L (*n* = 9345) | 42 | 1.00 (0.95, 1.06) |
| **Aspartate transaminase** |  |  |
| ≤ 39 U/L (*n* = 388,992) | 2145 | 0.94 (0.89, 0.99) |
| > 39 U/L (*n* = 16,421) | 83 | 1.25 (0.99, 1.59) |
| **Gamma-glutamyl transferase** |  |  |
| ≤ 64 U/L (*n* = 369,254) | 1997 | 1.07 (0.98, 1.17) |
| > 64 U/L (*n* = 36,159) | 231 | 0.99 (0.87, 1.12) |
| **Total bilirubin** |  |  |
| ≤ 17 μmol/L (*n* = 386,625) | 2142 | 0.88 (0.82, 0.94) |
| > 17 μmol/L (*n* = 18,788) | 86 | 1.25 (0.98, 1.58) |

^a^ adjusted for age, sex, ethnicity, BMI, smoking status, physical activity level, alcohol consumption, Townsend deprivation index, C-reactive protein, healthy diet and Charlson Comorbidity Index

Abbreviations: BMI, body mass index; CI confidence interval; HR, hazard ratio; IBD, inflammatory bowel disease; SD, standard deviation.

## Table S12 Association between MAFLD with different risk of advanced fibrosis and risk of IBD, CD and UC according to BAAT score ^a^

|  | **Incident IBD** | | |  | **Incident CD** | | |  | **Incident UC** | | |
| --- | --- | --- | --- | --- | --- | --- | --- | --- | --- | --- | --- |
|  | Cases/person-years | HR (95% CI) | *P* |  | Cases/person-years | HR (95% CI) | *P* |  | Cases/person-years | HR (95% CI) | *P* |
| **BAAT** | |  |  |  |  |  |  |  |  |  |  |
| Non-MAFLD | 1273/3,109,082 | Ref |  |  | 377/3,109,082 | Ref |  |  | 896/3,109,082 | Ref |  |
| MAFLD-not at high risk of advanced fibrosis | 105/224,621 | 1.07 (0.87, 1.32) | 0.500 |  | 41/224,621 | 1.58 (1.12, 2.22) | 0.008 |  | 64/224,621 | 0.89 (0.68, 1.15) | 0.37 |
| MAFLD-at high risk of advanced fibrosis | 850/1,582,534 | 1.12 (1.02, 1.23) | 0.013 |  | 267/1,582,534 | 1.32 (1.11, 1.55) | 0.001 |  | 583/1,582,534 | 1.05 (0.94, 1.17) | 0.40 |

^a^ adjusted for age, sex, ethnicity, smoking status, physical activity level, alcohol consumption, Townsend deprivation index, healthy diet and Charlson Comorbidity Index

Abbreviations: CD, Crohn's disease; CI, confidence interval; HR, hazard ratios; IBD, inflammatory bowel disease; MAFLD, metabolic dysfunction-associated fatty liver disease; UC, ulcerative colitis.

# Supplementary Methods

**Assessment on the risk of advanced fibrosis by BAAT score**

The risk of advanced fibrosis was assessed by using the BAAT score which was calculated by considering the parameters of body mass index (BMI), age, alanine aminotransferase, and triglycerides. Age-specific BAAT score was calculated for each variable according to the following formula: age (≥ 50 years = 1; < 50 years = 0), BMI (≥ 28 kg/m2 = 1; < 28 kg/m2 = 0), TG (≥ 1.7 mmol / L = 1; < 1.7 mmol/L = 0), ALT (≥ 2 times normal = 1; < 2 times normal = 0). The BAAT score ranged between 0 and 4, and a cut-off ≥ 2 was recommended for the definition of high risk of advanced fibrosis.

**Covariates**

**Age at recruitment**

This is a derived variable based on date of birth and date of attending an initial assessment centre and refers to the age of the participant on the day they attended an Initial Assessment Centre, truncated to whole year.

**Sex**

A mixture of the sex the National Health Service had recorded for the participant and self-reported sex. We treated it as a categorical variable (“Female”, “Male”).

**Ethnicity**

We used the self-reported answer to “What is your ethnic background”. We classified the responses into: White (White) and Others (Mixed, Asian or Asian British, Black or Black British, Chinese, and other ethnic group)

**Education**

We used the self-reported answer to “Which of the following qualifications do you have”. We classified the responses into: College (College or University degree) and Below college (A levels/AS levels or equivalent, O levels/GCSEs or equivalent, CSEs or equivalent, NVQ or HND or HNC or equivalent, other professional qualifications e.g.: nursing, teaching, and none of the above)

**Townsend deprivation index**

Townsend deprivation index was derived according to the unemployment rate, the percentage of overcrowded households, the percentage of people without cars, and the percentage of people without houses for each area in the UK, and baseline Townsend deprivation index calculated immediately before participant joining UK Biobank based on the preceding national census output areas. Each participant was assigned a score corresponding to the output area in which their postcode is located [9]. Higher scores represent the more socioeconomic deprivation one is suffering.

**Smoking status**

We used the self-reported answer to “current/past smoking status”. We classified the responses into: Never smoked (Never) and previous or current smoker (Previous, current)

**Alcohol consumption**

Participants reported the number of alcohol units (10 ml of pure ethanol) consumed, in “units per week” (for frequent drinkers) or “units per month” (for less frequent drinkers), across several beverage categories (red wine, white wine/champagne, beer/cider, spirits, fortified wine, and “other”). For each beverage, their standardised unit and corresponding drink-equivalents are presented as follow [10]:

| Beverage | Drink-equivalents (containing 14 g of pure alcohol) |
| --- | --- |
| 125 mLwine | 0.85 |
| 4% ABV pint beer | 1.28 |
| 25 mL spirits | 0.57 |
| 50 mL fortified wine | 0.56 |

None to moderate level of alcohol consumption was defined as 0–14 g/d for women and 0–28 g/d for men according to US dietary guidelines, above which is defined as heavy level [11]. And we named the two levels as “never or moderate” and “heavy” to avoid confusion.

**Physical activity level:**

At baseline, participants were asked a series of questions from the International Physical Activity Questionnaire (IPAQ) short form [12] regarding their normal daily activities. According to American Heart Association [13], physical activity was categorized into **Adequate** (150 minutes moderate activity per week OR ≥ 75 minutes vigorous activity per week OR equivalent combination OR moderate physical activity at least 5 days a week or vigorous activity once a week) and **Inadequate** (below adequate level).

**BMI**

BMI value is constructed from height and weight measured during the initial assessment centre visit. Relevant variable was measured by trained staff.

**Healthy diet**

Lourida I et al. [10] developed a variable evaluating whether participants have a healthy diet in the UK Biobank using food frequency questionnaire. food frequency questionnaire collected following items in either quantitatively or as frequency of intake (e.g., 2–4 times/week). For the variables recorded in the frequency, we assigned it with mean value get quantitative estimate (e.g., 2–4 times/week → 3 servings/week). A “healthy” diet met at least 4 criteria in the following 7 food groups:

| **Food groups** | Criteria |
| --- | --- |
| **Fruits** (items in FFQ: dried fruit, fresh fruit) | ≥ 3 servings/day |
| **Vegetables** (items in FFQ: cooked vegetable, raw or salad vegetable) | ≥ 3 servings/day |
| **Fish** (items in FFQ: oily fish, non-oily fish) | ≥2 servings/week |
| **Unprocessed red meats** (items in FFQ: unprocessed pork, beef, mutton) | ≤ 1.5 servings/week |
| **Whole grains** (items in FFQ: cereal intake, wholemeal or wholegrain bread) | ≥ 3servings/day |
| **Refined grains** (items in FFQ: other bread intake) | ≤1.5 servings/day |
| **Processed meats** (items in FFQ: processed meat) | ≤ 1 serving/week |

**C-reactive protein**

Assessed using immuno-turbidimetric method with platform (Beckman Coulter AU5800).

**Charlson Comorbidity Index (CCI)**

CCI is a highly cited and well-established tool for measuring comorbidity in clinical research. We calculated CCI as a variable reflecting objective heath status (range: 0–16). Mak JKL et al [14] developed the calculation of CCI in the UK Biobank, which were constructed based on 17 comorbidities (myocardial infarction, congestive heart failure, peripheral vascular disease, cerebral vascular disease, dementia, pulmonary disease, connective tissue disorder, peptic ulcer, liver disease, diabetes, diabetes complications, paraplegia, renal disease, cancer, metastatic cancer, severe liver disease, and acquired immune deficiency syndrome) with assigned weights associated with ICD codes from hospital records [14-16].

**Medication use**

*Non-steroidal anti-inflammatory drugs*

We used the self-reported answer to “Do you regularly take any of the following?” We classified the usage of non-steroidal anti-inflammatory drugs into: **Yes** (Who chose option “aspirin”, “Ibuprofen”, or “Paracetamol”) and **No (**other choices**)**. selection allowed for the usual names of the corresponding drugs will be verified in the verbal interview.

*Proton pump inhibitors*

We used the data from verbal interview by trained nurse on prescription medications. Data on any regular treatments taken weekly, monthly, etc. It does not include short-term medications (such as a 1week course of antibiotics) or prescribed medication that is not taken, or over-the-counter medications). For proton pump inhibitors, we classified the usage of proton pump inhibitors into: **Yes** (Who reported Lansoprazole, omeprazole, pantoprazole, esomeprazole, and rabeprazole) and **No (**other medicine) based on the previous study [17].

*Antibiotics*

We used the data from verbal interview by trained nurse on prescription medications. Data on any regular treatments taken weekly, monthly, etc. It does not include short-term medications (such as a 1week course of antibiotics) or prescribed medication that is not taken, or over-the-counter medications). For proton pump inhibitors, we classified the usage of proton pump inhibitors into: **Yes** (Tetracyclines, Beta-lactam antibacterials and penicillins, xulfonamides and trimethoprim, macrolides, lincosamides, and streptogramins, aminoglycoside antibacterials, quinolone antibacterials, other antibacterials) and **No (**other medicine) based on the previous study [18].

*Hormonal replacement therapy*

We used the self-reported answer to “Have you ever used hormone replacement therapy (HRT)?” We classified the usage of hormonal replacement therapy into: **Yes** (Who chose option “Yes”) and **No (**other choices**)**.

*Oral contraceptive pills*

We used the self-reported answer to “Have you ever taken the oral contraceptive pill? (include the 'mini-pill')” We classified the usage of oral contraceptive pills into: **Yes** (Who chose option “Yes”) and **No (**other choices**)**.

**Reference**

[1] Bedogni G, Bellentani S, Miglioli L, Masutti F, Passalacqua M, Castiglione A, et al. The Fatty Liver Index: a simple and accurate predictor of hepatic steatosis in the general population. BMC Gastroenterology. 2006;6:33.

[2] Castera L, Friedrich-Rust M, Loomba R. Noninvasive Assessment of Liver Disease in Patients With Nonalcoholic Fatty Liver Disease. Gastroenterology. 2019;156:1264-81.e4.

[3] Eslam M, Newsome PN, Sarin SK, Anstee QM, Targher G, Romero-Gomez M, et al. A new definition for metabolic dysfunction-associated fatty liver disease: An international expert consensus statement. Journal of Hepatology. 2020;73:202-9.

[4] Eastwood SV, Mathur R, Atkinson M, Brophy S, Sudlow C, Flaig R, et al. Algorithms for the Capture and Adjudication of Prevalent and Incident Diabetes in UK Biobank. PLoS One. 2016;11:e0162388-e.

[5] Carvalho-e-Silva AP, Harmer AR, Ferreira ML, Ferreira PH. The effect of the anti-diabetic drug metformin on musculoskeletal pain: A cross-sectional study with 21,889 individuals from the UK biobank. European Journal of Pain. 2021;25:1264-73.

[6] Yuan G, Al-Shali KZ, Hegele RA. Hypertriglyceridemia: its etiology, effects and treatment. CMAJ. 2007;176:1113-20.

[7] Liu Z, Suo C, Shi O, Lin C, Zhao R, Yuan H, et al. The Health Impact of MAFLD, a Novel Disease Cluster of NAFLD, Is Amplified by the Integrated Effect of Fatty Liver Disease–Related Genetic Variants. Clinical Gastroenterology and Hepatology. 2022;20:e855-e75.

[8] Shrestha S, Olén O, Eriksson C, Everhov Å H, Myrelid P, Visuri I, et al. The use of ICD codes to identify IBD subtypes and phenotypes of the Montreal classification in the Swedish National Patient Register. Scand J Gastroenterol. 2020;55:430-5.

[9] Blane D, Townsend P, Phillimore P, Beattie A. Health and Deprivation: Inequality and the North. British Journal of Sociology. 1987;40:344.

[10] Lourida I, Hannon E, Littlejohns TJ, Langa KM, Hyppönen E, Kuzma E, et al. Association of Lifestyle and Genetic Risk With Incidence of Dementia. Jama. 2019;322:430-7.

[11] US Department of Agriculture, US Department of Health and Human Services. Dietary Guidelines for Americans 2020-2025. https://www.dietaryguidelines.gov/sites/default/files/2020-12/Dietary_Guidelines_for_Americans_2020-2025.pdf. [accessed 23 March 2022]

[12] Craig CL, Marshall AL, Sjöström M, Bauman AE, Booth ML, Ainsworth BE, et al. International physical activity questionnaire: 12-country reliability and validity. Medicine & science in sports & exercise. 2003;35:1381-95.

[13] Lloyd-Jones DM, Hong Y, Labarthe D, Mozaffarian D, Appel LJ, Van Horn L, et al. Defining and setting national goals for cardiovascular health promotion and disease reduction: the American Heart Association's strategic Impact Goal through 2020 and beyond. Circulation. 2010;121:586-613.

[14] Mak JKL, Kuja-Halkola R, Wang Y, Hägg S, Jylhävä J. Frailty and comorbidity in predicting community COVID-19 mortality in the U.K. Biobank: The effect of sampling. J Am Geriatr Soc. 2021;69:1128-39.

[15] Deyo RA, Cherkin DC, Ciol MA. Adapting a clinical comorbidity index for use with ICD-9-CM administrative databases. J Clin Epidemiol. 1992;45:613-9.

[16] Quan H, Sundararajan V, Halfon P, Fong A, Burnand B, Luthi JC, et al. Coding algorithms for defining comorbidities in ICD-9-CM and ICD-10 administrative data. Med Care. 2005;43:1130-9.

[17] Xia B, Yang M, Nguyen LH, et al. Regular Use of Proton Pump Inhibitor and the Risk of Inflammatory Bowel Disease: Pooled Analysis of 3 Prospective Cohorts. Gastroenterology. 2021;161(6):1842-1852.e10.

[18] Nguyen LH, Örtqvist AK, Cao Y, et al. Antibiotic use and the development of inflammatory bowel disease: a national case-control study in Sweden. Lancet Gastroenterol Hepatol. 2020;5(11):986-995.
